# Severe adverse events during second-line tuberculosis treatment in the context of high HIV Co-infection in South Africa: a retrospective cohort study

**DOI:** 10.1186/s12879-016-1933-0

**Published:** 2016-10-21

**Authors:** Kathryn Schnippel, Rebecca H. Berhanu, Andrew Black, Cynthia Firnhaber, Norah Maitisa, Denise Evans, Edina Sinanovic

**Affiliations:** 1 Right to Care, Johannesburg, South Africa; 2 Clinical HIV Research Unit, School of Clinical Medicine, Faculty of Health Sciences, University of the Witwatersrand, Johannesburg, South Africa; 3 Health Economics Unit, School of Family and Public Health, Faculty of Health Sciences, University of Cape Town, Cape Town, South Africa; 4 Health Economics and Epidemiology Research Office, Department of Internal Medicine, School of Clinical Medicine, University of the Witwatersrand, Johannesburg, South Africa; 5 School of Medicine, University of North Carolina, Chapel Hill, USA; 6 Wits Reproductive Health and HIV Research Institute, School of Clinical Medicine, Faculty of Health Sciences, University of the Witwatersrand, Johannesburg, South Africa; 7 GlaxoSmithKline, Bryanston, South Africa

**Keywords:** HIV, Antiretroviral therapy, Multi-drug resistant TB, Tuberculosis, Adverse drug reactions

## Abstract

**Background:**

According to the World Health Organization, South Africa ranks as one of the highest burden of TB, TB/HIV co-infection, and drug-resistant TB (DR-TB) countries. DR-TB treatment is complicated to administer and relies on the use of multiple toxic drugs, with potential for severe adverse drug reactions. We report the occurrence of adverse events (AEs) during a standardised DR-TB treatment regimen at two outpatient, decentralized, public-sector sites in Johannesburg, South Africa.

**Methods:**

We reviewed medical records of the six-month intensive treatment phase for rifampicin-resistant (RR) TB patients registered May 2012 - December 2014. Patients contributed follow-up time until death, loss from treatment, censoring (6 months) or data extraction. A standardized regimen of kanamycin, moxifloxacin, ethionamide, terizidone, and pyrazinamide was used according to national guidelines. AEs were graded using the AIDS Clinical Trial Group scale. We present subhazard ratios from competing risk analysis for time to severe AE, accounting for mortality and loss from treatment.

**Results:**

Across the two sites, 578 eligible patient files were reviewed. 36.7 % were categorized as low weight (≤50 kg) at DR-TB initiation. 76.0 % had no history of TB treatment prior to the current episode of RR TB. 26.8 % were diagnosed with RR TB while hospitalized, indicating poor clinical condition. 82.5 % of patients were also HIV positive, of whom 43.8 % were on ART prior to RR TB treatment and 32.1 % initiated ART with or after RR TB treatment. Median CD4 count was 114.5 (IQR: 45-246.5). Overall, 578 reports of AEs were captured for 204 patients (35.3 %) and 110 patients (19.0 %) had at least one severe AE reported. Patients with at least one AE experienced a median of 3 (IQR: 2-4) AEs per patient. HIV-positive patients with CD4 counts ≤100 cells/mm^3^ and those newly initiating ART were more likely to experience a severe AE (sHR: 2.76, 95 % CI: 1.30–5.84 and sHR: 3.07, 95 % CI: 1.46–6.46, respectively).

**Conclusion:**

Severe AE are common during the first 6 months of RR TB treatment and HIV-positive patients newly initiating ART have the highest subdistribution hazard ratio for severe AE, accounting for the competing risks of death and loss from treatment.

## Background

South Africa has made progress in controlling the TB epidemic; the 2015 Global Tuberculosis Report highlights a declining incidence and prevalence of TB for South Africa [1]. Despite the progress seen tuberculosis has been the most common cause of death in South Africa from 2005 to 2014 [2] and the number of persons diagnosed with drug-resistant (DR-TB) TB has increased significantly over the last decade from 2000 patients in 2005 to 18,734 in 2014 [1].

In 2011, South Africa adopted a policy of universal rifampicin (RIF) resistance testing using Xpert MTB/RIF (Cepheid, USA) as the first-line TB diagnostic in the country. Reporting of DR-TB now focuses on RIF resistant TB (RR TB), which includes RIF resistance with unknown or pending sensitivities to other drugs, mono-RIF resistant TB, multi-drug resistant (MDR) TB that is resistant to both RIF and isoniazid (INH), extensively drug resistant (XDR) TB which is MDR TB plus resistance to second-line drugs from the fluoroquinolone and injectable aminoglycoside or cyclic peptide classes, or preXDR TB which is MDR TB plus resistance to either a fluoroquinolone *or* a second-line injectable drug [3]. The South African National TB Programme (NTP) treatment guidelines for RR-TB indicate 18‐24 months of treatment [4]. All RR TB patients are started on the standardized second-line MDR TB regimen until further resistance is either confirmed or ruled-out at which point the patient may be switched to an individualized second-line TB regimen (if preXDR or XDR TB) or INH may be added to the regimen (if mono-RIF resistant) [4].

Second‐line TB treatment is complicated to administer, with frequent and potentially severe adverse drug reactions (ADR) [5, 6]. Adding to the complexity of treatment, an estimated 60 % of all TB patients [1] and up to 80 % of RR-TB patients in South Africa [7] are also HIV-infected and therefore eligible for antiretroviral therapy (ART). RR TB treatment and ART present overlapping toxicities which may be worsened by concomitant use, for example both kanamycin and tenofovir may cause renal dysfunction [8].

ADR can negatively impact the effectiveness of RR TB treatment in many ways. Patients or clinicians may interrupt, reduce dosage, or stop treatment in an attempt to alleviate side effects. This results in an increased risk of acquiring additional drug resistance, failing treatment, or dying from TB. ADR themselves may also result in hospitalization, permanent disability, or death. Only 49 % of the 2012 cohort of MDR TB patients in South Africa were cured or successfully completed treatment, below both the global average (50 %) and targets (75 %) [1]. Thus, evidence of the burden and risks of ADR during RR TB treatment is important for both patients and clinicians to manage this complexity. In order to quantify the burden of ADR during outpatient RR TB treatment in the context of high co-infection with HIV, we present the results of a medical file review of routinely reported adverse events (AE) from two decentralised public-sector sites within South Africa.

## Methods

The study was conducted at the TB Focal Point clinics at Helen Joseph and Charlotte Maxeke Academic Hospitals in Johannesburg, South Africa. Patients with laboratory diagnosis of pulmonary or extrapulmonary RR TB from any level of health facility in the catchment area are referred to these NGO-supported, outpatient, public-sector facilities for initiation of second-line TB treatment and further drug resistance testing. Patients are referred from surrounding primary health care centres, private facilities, Helen Joseph and Charlotte Maxeke inpatient wards, and surrounding hospitals. A census of medical files for patients who enrolled for second-line TB treatment at Helen Joseph (May 2012 to June 2014) or Charlotte Maxeke (May 2012 to December 2014) were reviewed retrospectively in September 2014 and April 2015, respectively. Only patients with documentation of at least rifampicin resistance, i.e. RIF resistance detected by Xpert, RIF mono-resistance, MDR-TB diagnosed by LPA, or XDR TB were included in the study.

### Standard of care treatment

Upon referral the patient undergoes HIV counselling and testing, patients are educated about the length and toxicities of RR TB treatment, and informed about the importance of screening exposed family members. Patients are then examined and evaluated by a medical officer. All RIF resistance diagnosed by Xpert is treated presumptively as MDR TB as per the South African National TB Programme (NTP) guidelines [4]. The intensive phase of treatment (approximately 6 months) consists of a five-drug regimen including the second-line injectable kanamycin, moxifloxacin, ethionamide, terizidone, and pyrazinamide [4, 9]. The continuation phase of treatment (18 to 24 months) includes moxifloxacin, ethionamide, terizidone, and pyrazinamide. Treatment dose is adjusted for patient weight, with patients 33 to 50 kg receiving smaller doses of kanamycin (500–750 mg vs. 1000 mg), ethionamide (500 mg vs. 750 mg), and pyrazinamide (1000–1750 mg vs. 2000–2500 mg) than patients weighing 51 to 70 kg [4]. Linezolid and bedaquiline were not available at the study sites during the study period [10].

The 2010 South African ART guidelines indicated eligibility for HIV-infected patients with TB co-infection at CD4 ≤ 350 cells mm^3^ and for any patient with CD4 ≤ 200 cells mm^3^ [11]. Eligibility was expanded in 2013 to include all patients with CD4 count ≤350 cells mm^3^ [12]. Thus, all RR TB patients not already on ART are initiated at the RR TB site. Patients currently on ART can receive ART from the RR TB site for the duration of second-line TB treatment. From 2010, most South African ART patients in the public sector are initiated on a standard, first-line three-drug regimen of tenofovir, efavirenz or nevirapine, and lamivudine or emtricitabine. From 2013, a fixed-dose combination of tenofovir, efavirenz, and emtricitabine has been the preferred first-line ART regimen [12].

Patients are asked to return at two weeks and subsequently reviewed monthly by a TB nurse or medical officer for adherence to therapy, evaluation of side effects and treatment response. Sputum samples for smear microscopy and TB culture are collected monthly in pulmonary TB patients to assess for sputum smear and culture conversion. As per NTP treatment guidelines, baseline laboratory tests are conducted prior to treatment initiation to assess for anaemia and renal, thyroid and liver function. If HIV co-infected, laboratory tests to monitor HIV treatment (CD4 count and HIV viral load) or ART toxicities are integrated. Every two months during the six-month intensive phase of treatment blood work is repeated. Additional or more frequent laboratory testing may be ordered if clinically indicated. Audiology testing for hearing loss is conducted monthly during the six month intensive phase due to the high rates of aminoglycoside induced hearing dysfunction [13]. Electrocardiograms for monitoring were not included in the guidelines for the standard RR TB regimen at the time of the study.

### Adverse events and drug reactions

For most of the period under review, pharmacovigilance requirements were for targeted spontaneous reporting to the national regulatory agency, the Medicines Control Council. Programmatic pharmacovigilance for HIV and TB, including RR TB was in the process of being rolled out by the National Pharmacovigilance Centre [14]. According to the regulatory form which is separate from the medical file, ‘advice about voluntary reporting’ requests clinicians to please report all ‘serious reactions and interactions with all products’ to the Medicines Control Council.

Most of the AEs identified in this file review were found in the clinical progress notes section of the medical files. Causality was not routinely documented. AEs such as weight loss could be related to adverse drug reactions from second-line TB treatment or ART, or HIV or TB morbidity. Pre-existing conditions at the time of RR TB treatment initiation were noted under patient medical history and co-morbidities and therefore not included in the adverse events. However, if pre-existing conditions worsened during treatment it would be reported.

AEs of interest were those most likely to be adverse drug reactions and defined prior to the review as including renal dysfunction, hypokalaemia, ototoxicity, vestibular dysfunction, severe anaemia, psychosis and depression, peripheral neuropathy, seizures, hypothyroidism, nausea and/or vomiting, and joint pain. Other AEs identified during the review were recorded and categorized during data analysis. Standard of care does not include routine screening for gastro-intestinal disorders such as nausea or vomiting or mental health such as depression, or insomnia; AEs captured were as reported by patients during routine visits.

AEs were graded either at the time of the event (in the clinical notes) or during the file review by the treating clinician as mild (grade 1), moderate (grade 2), severe (grade 3), potentially life-threatening (grade 4), or fatal (grade 5) according to the AIDS Clinical Trials Group grading system [15]. AEs included those detected clinically and through laboratory testing. Where outcome of the AE (e.g. death, hospitalization, permanent disability, drug discontinuation, and/or drug dose reduction) was available in the file it was also captured. Deaths that were not reported as an outcome of an AE, i.e. those thought to be from TB, were not re-categorised as an AE.

### Statistical analysis

We present descriptive analysis of the number of AEs by patient, types of AE reported, and severity of AEs identified from the medical record review, including frequencies and proportions.

We used time to event analysis to present the mean time from second-line TB treatment initiation to (first) severe adverse event (grade 3 or higher). Follow-up is censored at 6 months after second-line TB treatment initiation, final outcome (i.e. death or loss from treatment for at least 2 months), transfer to another site, or data extraction (September 2014 for Helen Joseph and April 2015 for Charlotte Maxeke).

We used competing risk regression method from Fine and Gray [16] to determine if occurrence of a severe AE was associated with *a priori* identified patient demographic and clinical characteristics. In time-to-event analysis, an individual can either experience the event of interest or be censored. However, in real life, an individual may experience a number of negative outcomes, not only the event of interest. Occurrence of one negative outcome means that the other negative outcomes cannot occur, and only the time to failure for the earliest event is observed. The method of competing risk regression was chosen because of the high rate of mortality and loss from treatment in South African RR TB cohorts; one of these poor outcomes could occur before the severe AE and therefore the severe AE would not occur (or would occur but never be documented). Because loss from treatment may represent unreported mortality, both death and loss from treatment were considered to be the risks competing with report of a severe AE. Univariate (crude) subdistribution hazard ratios (sHR) with 95 % confidence intervals (CI) are presented. All analysis was completed in Stata v14 (College Station, TX).

Characteristics considered included: HIV status (HIV negative, HIV-positive on ART prior to RR TB diagnosis and initiation defined as at least 30 days prior to RR TB treatment start, HIV-positive initiated on ART at or after RR TB initiation, or HIV positive not on ART or ART unknown); age category (10–24, 25–39, 40–54, 55 years or older), and sex. We also examined TB symptoms (report of cough, fever, weight loss, or night sweats), and markers of severity of illness: low weight at treatment initiation (≤50 kg) or diagnosed with RR TB during hospitalization for any cause. CD4 count at RR TB treatment initiation as documented in the patient file (HIV negative, HIV-positive with CD4 > 100 cells/ mm^3^, or HIV-positive with CD4 ≤ 100 cells/mm^3^) was defined as the CD4 count measured up to 6 months prior or within the first month of treatment. Information on pre-existing conditions or self-reports of prior TB treatment (none, first-line regimen, or regimen that included streptomycin) were also considered. No patients reported having been previously treated for RR TB. All patients were on standardized treatment regimens for RR TB; patients diagnosed with XDR TB were transferred out to initiate individualized treatment at another site and therefore exposure was censored.

## Results

Across the two sites, 578 files were available for review and patients eligible for the study, patient characteristics are reported in Table 1. Patients presented with RR TB at a median age of 35 (IQR: 29–42 years) and 49.0 % were male. One-third of the patients (36.7 %) were categorized as low weight (≤50 kg) at initiation of second-line TB treatment, the median weight at initiation was 54 kg (IQR: 47.9–61.5). Only 1.2 % reported a history of TB treatment with an injectable drug (streptomycin) and the majority (76.0 %) had no history of any TB treatment prior to the current episode of RR TB. Approximately one-quarter (26.8 %) of the patients were diagnosed with RR TB while hospitalized, indicating poor clinical condition.

**Table 1:** Characteristics of patients at initiation of second-line TB treatment (*n* = 578)

Characteristic	Description	Count	Proportion
Sex	Male	283	49.0 %
	Female	295	51.0 %
Age	10–24 years	56	9.7 %
	25–39 years	338	58.5 %
	40–54 years	163	28.2 %
	55+ years	21	3.6 %
Weight (kg)	Low weight (≤50 kg)	195	36.7 %
	>51 kg	337	63.3 %
	Missing	46	8.0 %
TB foci	Pulmonary	541	93.6 %
	Extrapulmonary only	37	6.4 %
Prior TB	No history of TB treatment	439	76.0 %
	Prior first-line TB treatment	119	20.6 %
	Prior TB treatment with streptomycin	7	1.2 %
	Unknown	13	2.2 %
Current TB diagnosis	MDR-TB (INH and RIF resistance)	182	31.5 %
	RIF mono-resistant TB	198	34.3 %
	RIF resistant, sensitivities unknown	191	33.0 %
	XDR TB (second-line resistance)	7	1.2 %
Presenting symptoms	Cough	281	65.9 %
	Any of cough, weight loss, fever, night sweats	453	78.4 %
Sputum smear microscopy	Positive (scanty or higher)	284	49.1 %
	Negative or unknown	294	50.9 %
Level of care at TB diagnosis	Outpatient, ambulatory	406	70.2 %
	Inpatient, hospitalized	155	26.8 %
	Missing	17	2.9 %

*MDR-TB* multi-drug resistant tuberculosis, *RIF* rifampicin, *INH* isoniazid, *XDR TB* extensively drug-resistant tuberculosis

HIV infection was the most common co-morbidity reported as 82.5 % (*n* = 477/578) were HIV positive (Table 2). Of those who were HIV positive, 43.8 % were on ART at least 30 days prior to RR TB treatment initiation with a median 332 days on ART (IQR: 160, 991) and 31.9 % initiated ART with or after RR TB treatment with a median time to ART initiation of 26 days after RR TB treatment (IQR: 14, 42). Of the 77.8 % of HIV-positive patients with CD4 counts reported at RR TB treatment initiation, the median CD4 was 114.5 (IQR: 45-246.5). Half (50.2 %) were on the standard first-line regimen for the public sector ART program (tenofovir, efavirenz, and lamivudine or emtricitabine). Few other co-morbidities were reported, with no one co-morbidity affecting 10 or more patients. Diabetes mellitus (1.9 %), renal insufficiency (1.4 %), and hepatitis or liver dysfunction (1.2 %) were the three most common co-morbidities other than HIV. Seven women (2.4 % of women) were pregnant during RR TB treatment.

**Table 2:** Co-morbidities, clinical conditions and chronic medications (*n* = 578)

Characteristic	Description	Count	Proportion
HIV status	Negative	95	16.4 %
	Positive	477	82.5 %
	Unknown	6	1.0 %
CD4 count^a^ (*n* = 477)	Low (≤100 cells/mm^3^)	173	36.3 %
	>100 cells/mm^3^	198	41.5 %
	Missing	106	22.2 %
ART status (*n* = 477)	Not on ART	116	24.3 %
	Median CD4 count	156.5	IQR: 65, 255
	Initiated ART with or after RR TB	153	32.1 %
	Median CD4 count	100.5	IQR: 42.5, 221.5
	Median days RR TB at ART initiation	26	IQR: 14, 42
	On ART prior to RR TB initiation	209	43.8 %
	Median CD4 count	101	IQR: 41, 253
	Median days on ART at RR TB initiation	332	IQR: 160, 991
ART regimen (*n* = 362)	TDF + 3TC or ETC + EFV	182	50.23 %
	D4T or AZT + 3TC + EFV	34	9.4 %
	TDF + 3TC + LPV/r	3	0.8 %
	D4T or AZT + 3TC + LPV/r	46	12.7 %
	Other regimen	53	14.6 %
	Missing	44	12.2 %
Reported co-morbidities	Hepatitis or liver disorder	7	1.2 %
	Epilepsy	6	1.0 %
	Psychiatric disorder	5	0.9 %
	Diabetes mellitus	9	1.6 %
	Renal dysfunction	8	1.4 %
Pregnancy (*n* = 295)	Pregnant	7	2.4 %
Contraception (*n* = 295)	Using hormonal contraceptive	16	5.4 %

*ART* antiretroviral therapy, *TDF* tenofovir, *3TC* lamivudine, *EFV* efavirenz, *LPV/r* lopinavir/ritonavir

^a^CD4 count at RR TB treatment initiation

### Adverse events

Overall, 578 reports of AEs were captured for 204 patients (35.3 %) and 110 patients (19.0 %) had at least one severe AE (grade 3+). Patients with at least one reported AE experienced a median of 3 AEs (IQR: 2–4) per patient.

**Fig. 1:**
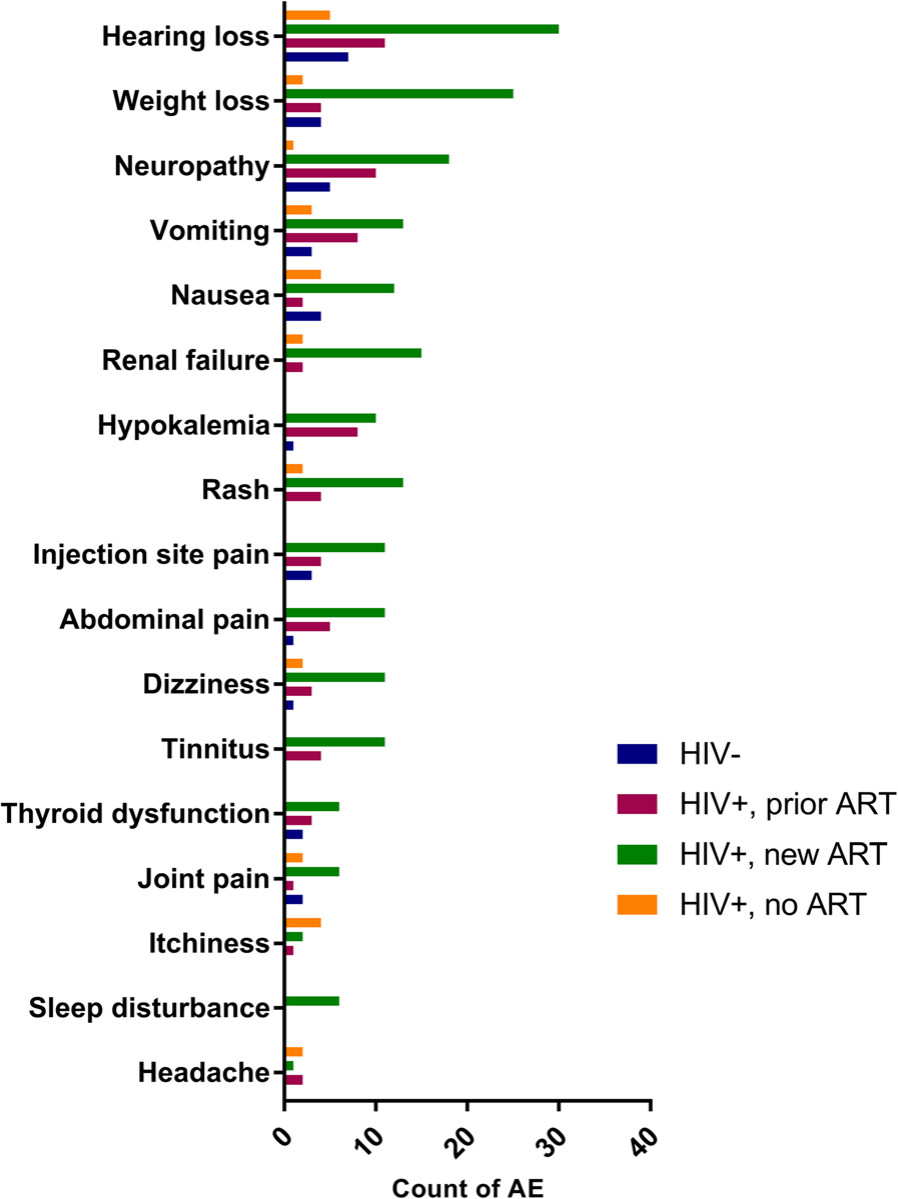
Counts of most frequent mild or moderate adverse events by HIV and ART status (*n* = 204)

**Fig. 2:**
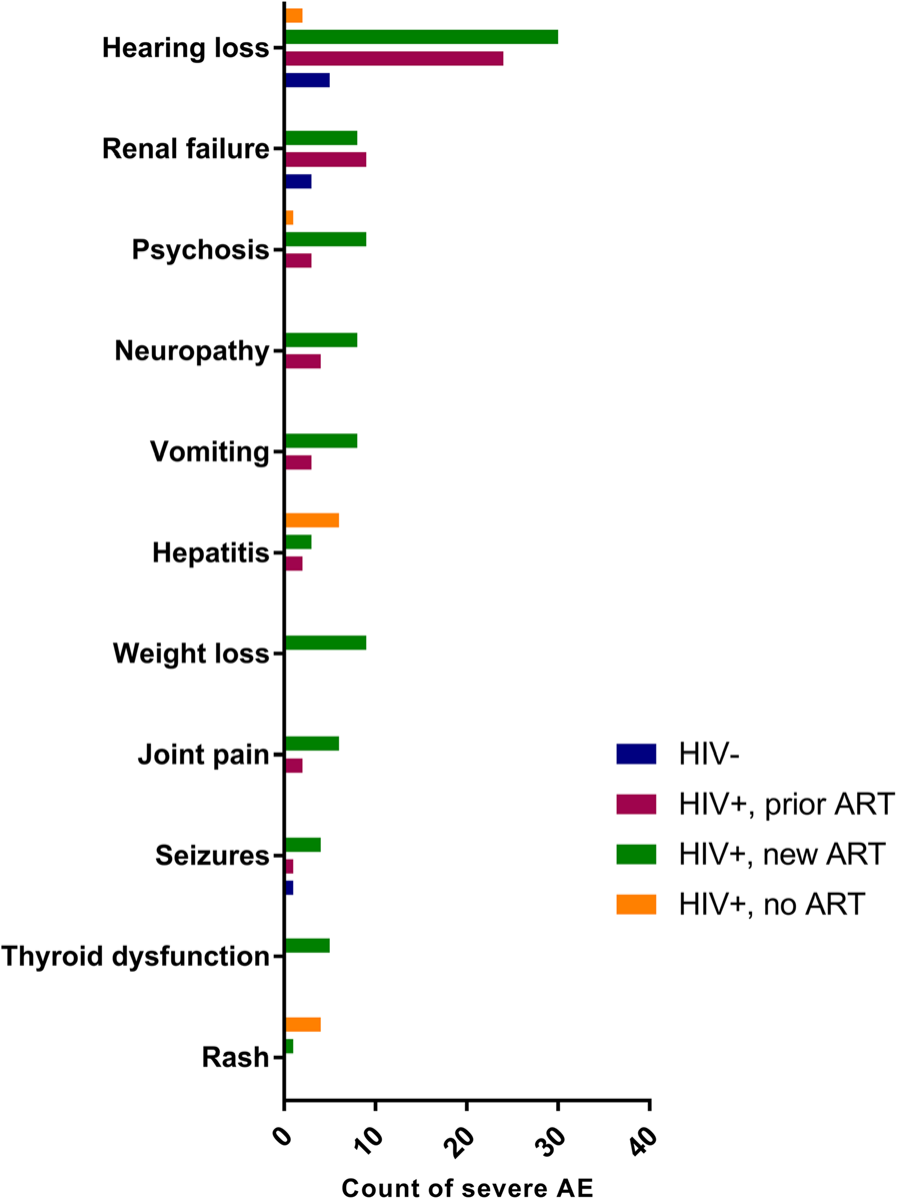
Counts of most frequent severe adverse events by HIV and ART status (*n* = 110)

Gastro-intestinal AEs were the most common, with 138 reports of vomiting, nausea, abdominal pain, epi-gastric discomfort, diarrhoea, constipation, loss of appetite, or weight loss. Although most nausea and vomiting reported (67.1 % of 70 reports) was mild to moderate (Fig. 1), it was the second most commonly reported severe AEs (11.8 % of all the severe AEs). The most common severe AE reported was hearing loss or ototoxicity (Fig. 2). In total, 114 reports of hearing loss were noted affecting 17.3 % of patients (*n* = 100/578). Of the hearing loss AEs reported, 61 (53.5 %) were categorised as grade 3+. Renal dysfunction or failure accounted for 10.3 % of all severe AEs, with 20 episodes reported. Psychosis (6.7 %), neuropathy (6.1 %) and hepatitis or liver dysfunction (5.6 %) were also among the most frequent severe AEs reported. Rare severe AE reports included deep vein thrombosis (*n* = 2), sepsis (*n* = 1), miscarriage (*n* = 1), suicidal thoughts (*n* = 1), and neuroleptic malignant syndrome (*n* = 1).

Kanamycin was listed as the suspected drug causing AE or severe AE for 54.4 % (111/204) of patients experiencing an AE. Among the 309 AEs with at least one suspected drug listed, kanamycin was listed 175 times (56.6 %). Of the severe AEs with at least one suspected drug listed, terizidone was listed 32/126 times (25.4 %). Less than 5 % (*n* = 26) of the AEs had hospitalization documented as an outcome of the AE. Nearly 20 % (*n* = 109) of identified AEs resulted in the suspected drug being discontinued and an additional 10 % (*n* = 57) resulted in the dose of the suspected drug being reduced.

### Subdistribution hazard ratios of severe AE

Of the 578 patient files reviewed, 18 did not contribute to the time-to-event analysis as patient either died or were transferred out prior to returning to the clinic after RR TB treatment initiation. The 560 that were included contributed 52,684 person-days of follow-up with a median exposure of 70 days (IQR: 28, 183) from treatment initiation until severe AE or censoring. Of the 560, 268 (47.9 %) completed 6 months of treatment, 171 (30.5 %) were followed until transferred to another site, 73 (13.0 %) were lost from treatment, and 48 (8.6 %) died. There were 107 severe AEs analysed, giving an incidence rate for severe AE of 0.74 per person year.

HIV-positive patients with low CD4 counts (≤100 cells/mm^3^) and those who initiated ART with RR TB treatment both were approximately 3 times more likely to experience a severe AE with crude sHR: 2.76 (95 % CI: 1.30–5.84) and sHR: 3.07 (95 % CI: 1.46–6.46), respectively (Table 3). Patients previously treated for TB with a regimen including streptomycin were also more likely to experience a severe AE, sHR: 3.49 (95 % CI: 1.52–8.02), all were ototoxicity. Subdistribution hazard ratios for categorized age, low weight at treatment initiation, smear microscopy positive, recent hospitalization, sex, cough at initiation, or pre-existing renal, liver, or psychiatric conditions were not statistically different.

**Table 3:** Risks of severe (grade 3+) adverse events during first 6 months of RR TB treatment

Characteristic	Description	sHR^a^	95 % CI
Age category	10–24 years	0.70	0.31–1.58
	25–39 years	Referent	
	40–54 years	0.92	0.61–1.39
	55 years +	1.93	0.85–4.37
HIV and CD4 status	HIV negative	Referent	
	HIV+, CD4 > 100 cells/mm^3^	1.81	0.84–3.89
	HIV+, CD4 ≤ 100 cells/mm3	**2.76**	**1.30–5.84**
HIV and ART status	HIV negative	Referent	
	HIV+, initiated ART prior to RR TB	1.77	0.83–3.77
	HIV+, initiated ART with or after RR TB	**3.07**	**1.46–6.46**
	HIV+, not on ART	1.15	0.43–3.10
Weight (kg)	Weight >51 kg	Referent	
	Low weight (≤50 kg)	1.43	0.97–2.10
Prior TB treatment	No TB history reported	Referent	
	History of first-line TB treatment	1.33	0.85–2.07
	History of streptomycin for TB treatment	**3.49**	**1.52–8.02**
Referring site	Outpatient facility	Referent	
	Inpatient facility	1.11	0.74–1.70
Sex	Female	Referent	
	Male	0.83	0.57–1.22
Smear microscopy	Sputum smear negative or not reported	Referent	
	Sputum smear positive (scanty or higher)	1.00	0.69–1.47
Presenting symptom	No cough	Referent	
	Any cough	1.37	0.90–2.08
Co-morbidities^b^	No reported pre-existing renal insufficiency, liver or psychiatric disorder	Referent	
	Pre-existing renal, liver, or psychiatric condition	0.47	0.11–1.93

Bolded values are statistically significant at *p*-value < 0.05

*ART* antiretroviral therapy, *RR TB* rifampicin resistant tuberculosis, *sHR* subdistribution hazard ratio

^a^sHR crude analysis from competing risk regression accounting for death and loss from treatment

^b^No pregnant women had a documented severe AE prior to censoring

**Fig. 3:**
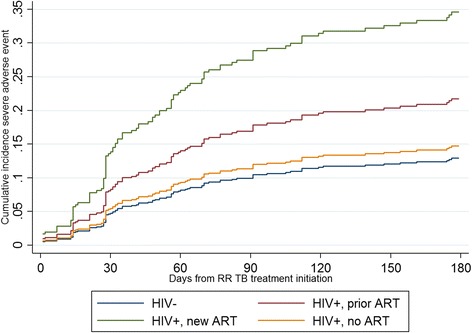
Cumulative incidence function after competing risk regression of any severe adverse event. Legend: Competing risk accounting for loss from treatment and death during treatment, by HIV and ART status

**Fig. 4:**
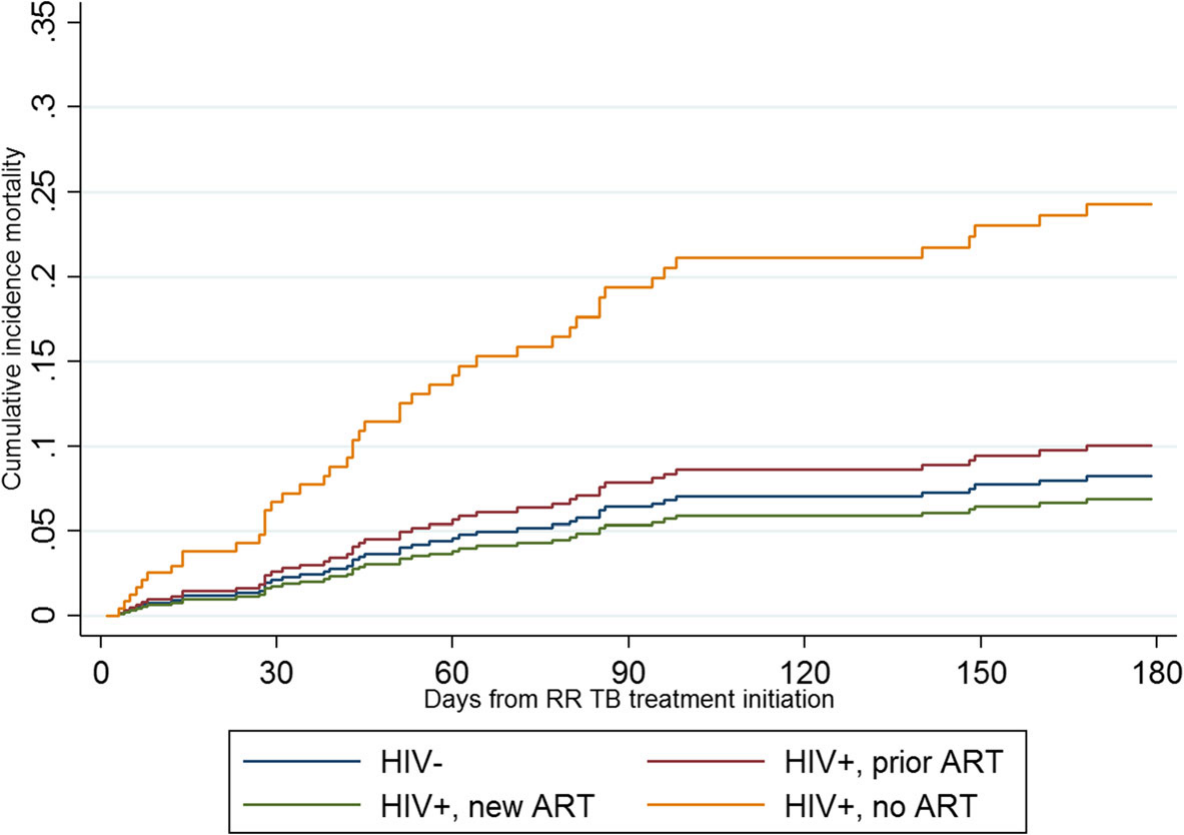
Cumulative incidence function after competing risk regression of death during RR TB treatment. Legend: Competing risk accounting for loss from treatment, by HIV and ART status

Competing risk regression sHR can be displayed as a graph of the cumulative incidence (of the risk analysed) function over the time at risk. Figure 3 shows patients initiating ART with or after RR TB treatment had the highest sHR of experiencing a severe AE versus HIV-negative patients, HIV-positive patients who had been on ART prior to RR TB diagnosis, and HIV-positive patients never on ART. As a comparison, the cumulative incidence function of death during RR TB treatment, accounting for the competing risk of loss from treatment, is shown for the same HIV and ART categories (Fig. 4). HIV-positive patients who did not initiate ART either before or at RR TB initiation were more than 3 times more likely to die during RR TB treatment than HIV-negative patients (sHR: 3.25, 95 % CI: 1.17–9.02). Figure 4 shows the cumulative incidence function for mortality during second-line TB treatment was highest for HIV-positive patients never on ART versus HIV-negative, HIV-positive patients who had been on ART prior to RR TB diagnosis, and HIV-positive patients initiating ART along with or after RR TB treatment.

## Discussion

During the first six months of RR TB treatment in a context where 82.5 % of patients were also infected with HIV, 35.3 % of patients experienced at least 1 AE. This incidence was less than reported in a meta-analysis of adverse drug events during the 18 to 24 months of treatment of MDR-TB, which found 57.3 % of patients (260/534) experienced at least one AE [6]. One of the included studies, from a similar context in South Africa, reported that 99 % of the 71 patients in that cohort experienced at least one AE [17]. The meta-analysis did not report whether the AEs were mild, moderate, severe, life threatening, or fatal [6]. In our cohort, the incidence of severe AEs during the first 6 months of treatment was 19.0 %, far higher than the 6.9 % reported in a large cohort of HIV positive, ART naïve MDR TB patients also from South Africa [18].

Kanamycin was listed as the suspected drug causing AE or severe AE for 54.4 % of cohort patients experiencing an AE. Kanamycin, along with other aminoglycosides, is associated with ototoxicity (hearing loss and vestibular dysfunction) and renal dysfunction [4, 13, 18, 19]. While an effective agent against drug-resistant TB [9], multiple trials are underway to develop an injection-free regimen for treating RR TB in an attempt to reduce the overall burden of ADR and improve adherence to long-term therapy [20].

Our finding that patients recently initiating both ART and RR TB treatment have an increased sHR for severe AE during the first 6 months (sHR: 3.07) has not been previously reported. The WHO recommendation to immediately initiate ART was based upon lower risk of death but found that there was ‘very low quality of evidence’ as to whether concomitant use of ART and RR TB treatment led to more severe AE or drug interactions [9]. A previous systematic review of the use of ART during second-line TB treatment concluded there was insufficient data as to whether concomitant use of ART and RR TB treatment increased the risk of ADR [8]. A study of XDR TB patients in South Africa found that there was no difference in the proportion of patients (using chi-squared test) with a severe AE reported for HIV-positive patients on ART compared to HIV-negative patients [21]. Our finding of the higher sHR for severe AE may be because other studies did not differentiate the grade of AE, had insufficient numbers of patients on ART, or did not differentiate the time on ART. Additionally, using the competing risk methodology to calculate sHR accounting for the competing risk of death and loss from treatment was useful in the context of very high early mortality for patients not on ART. The increased sHR point to a need for additional or more frequent monitoring for AE for patients initiating both ART and RR TB treatment at the same time. These patients may also benefit from inpatient treatment initiation where they can be more closely monitored and managed.

The cumulative incidence function for mortality accounting for the competing risk of loss to treatment indicated that patients not on ART are most at risk of death and therefore our results are consistent with studies and guidelines that indicate early initiation of ART for patients with TB [22–24] and drug-resistant TB [8, 9]. This reversal of risk (patients with highest relative sHR of mortality have the lowest relative sHR of severe AE) also highlights the need for greater access to ART and initiation at higher CD4 counts. In our study, there was a high proportion of patients with very low CD4 counts not on ART despite increasing access to ARVs in South Africa; patients with CD4 ≤ 100 cells/mm^3^ also had a higher sHR of severe AE (sHR: 2.76). Further, a quarter of the patient files reviewed in this study were diagnosed with RR TB while hospitalized although testing for RIF resistance is available at the lowest levels of the public health system in South Africa. Again, this represents delays in diagnosis and case finding at the primary health care level leading to hospitalization and late diagnoses.

While most nausea and vomiting were reported as mild to moderate (67.1 % of 70 reports) this AE can impact on both adherence and effectiveness of treatment. Because of AEs, the treating clinicians discontinued or reduced the dosage of the suspected drug for 17.3 % of patients (100/578), a similar proportion to the 21 % calculated from a patient-level meta-analysis of MDR-TB studies [25]. Because of overlapping toxicities, a patient who experienced an AE during RR TB treatment may no longer be eligible for standard ART or second-line TB treatment regimens, for example because of drug-induced liver or kidney injuries. Having fewer effective drugs in the second-line TB regimen is associated with lower probability of treatment success [25] and consequently higher risk of acquired additional resistance.

Prior exposure to streptomycin may be a risk factor for ototoxicity during RR TB treatment with increased aminoglycoside cumulative dose [13]. In our cohort, although the numbers of patients with prior exposure were small (1.2 %), this was the strongest predictor of a severe AE (sHR: 3.30), with all severe AEs related to ototoxicity. With the introduction of Xpert MTB/RIF diagnosis, the ‘retreatment’ regimen containing streptomycin was phased out and as of end 2013 is no longer in use in the South African NTP [26]. While the lack of history of prior TB treatment may reduce the risk of some types of ADR, from a public health perspective the 76.0 % of patients in this cohort without any prior TB history is concerning as it indicates primary transmission of RR TB.

### Limitations

The lower rates of ADR in this cohort may be a result of both the lack of routine reporting and retrospective study design. During the monthly visits to the outpatient clinic, patients are not prompted to self-report AE. In some cases, patients may have discussed AE with the counsellor, nurse, or clinician but if the healthcare worker did not note the discussion in the medical file it would not have been counted in this review. This may have resulted in an under-reporting of AEs, particularly those that were mild to moderate (grade 1-2). Conversely, clinicians may have spent more time monitoring or documenting AEs for patients who initiated treatment in poor clinical condition (e.g. hospitalized or concurrently starting on ART) compared to patients who were clinically stable.

Also, while guidelines indicate monitoring for certain AEs, implementation may differ from intended practice. One challenge that both sites noted during out study was that it was difficult to access audiology screening for patients due to concerns about infection control as audiology services were not co-located within the TB clinic. Additionally, our study focused on the first 6 months of treatment, the intensive phase, which includes kanamycin. It is possible that some patients who did not experience an ADR during the first 6 months would be affected in subsequent months or that an ADR would be detected in subsequent months, specifically aminoglycoside ototoxicity is known to occur even after treatment discontinuation [27].

Another limitation of this study was the inability to distinguish between the AE and ADR. For this reason, events described in this analysis were described as AE rather than the more specific term ADR. This limitation is common to studies describing ADR and AE and must be taken into consideration when trying to draw conclusions across studies. For example, in the South African study indicated above with 99 % incidence of AE, injection site pain was the second most commonly reported clinical AE. Injection site pain was only reported in this cohort for patients refusing to continue with the injections for reason of pain. Deaths were only included in the analysis if the clinician indicated that the death was likely due to an ADR; it is possible that deaths from ADR are underestimated as a result. Cause of death, particularly for outpatient care, are often not available as the patients die at home or in a different facility. This study also did not try to distinguish whether the AE was related to the ART or second-line TB treatment.

Finally, the study period preceded the roll-out of bedaquiline and linezolid in the South African NTP. These two drugs are now available for patients who experience toxicity to one of the drugs of the standard regimen [28] and use of the new and re-purposed drugs may affect the profile of ADR experienced by patients with drug-resistant TB in South Africa.

## Conclusions

Severe adverse events are common during the first 6 months of second-line treatment and HIV-positive patients newly initiating ART have the highest relative subdistribution hazard ratio of experiencing a severe AE, accounting for the competing risk of death and loss from treatment. Patients whose clinical condition requires immediate and concomitant initiation of ART and RR TB treatment may benefit from new RR TB treatment regimens that are better tolerated, intensive monitoring, or even inpatient treatment initially to watch for severe AEs.
